# Ciliary Muscle Dimension Changes With Accommodation Vary in Myopia and Emmetropia

**DOI:** 10.1167/iovs.63.6.24

**Published:** 2022-06-24

**Authors:** Dinesh Kaphle, Katrina L. Schmid, Leon N. Davies, Marwan Suheimat, David A. Atchison

**Affiliations:** 1 Centre for Vision and Eye Research, Queensland University of Technology, Kelvin Grove, Queensland, Australia; 2 Discipline of Optometry, Faculty of Health, University of Canberra, Bruce ACT 2617, Australia; 3 School of Optometry, College of Health and Life Sciences, Aston University, Birmingham, United Kingdom

**Keywords:** myopia, emmetropia, accommodation, ciliary muscle, optical coherence tomography

## Abstract

**Purpose:**

The purpose of this study was to determine whether accommodation-induced changes in ciliary muscle dimensions vary between emmetropes and myopes, and the effect of the image analysis method.

**Methods:**

Seventy adults aged 18 to 27 years consisted of 25 people with emmetropia (spherical equivalent refraction [SER] +0.21 ± 0.36 diopters [D]) and 45 people with myopia (−2.84 ± 1.72 D). There were 23 people with low myopia (>−3 D) and 22 people with moderate myopia (−3 to −6 D). Right eye ciliary muscles were imaged (Visante OCT; Carl Zeiss Meditec) at 0 D and 6 D demands. Measures included ciliary muscle length (CML), ciliary muscle curved length (CMLarc), maximum ciliary muscle thickness (CMTmax), CMT1, CMT2, and CMT3 (fixed distances 1–3 mm from the scleral spur), CM25, CM50, and CM75 (proportional distances 25%–75%). Linear mixed model analysis determined effects of refractive groups, race, and demand on dimensions. Significance was set at *P* < 0.05.

**Results:**

Myopic eyes had greater CML and CMLarc nasally than emmetropic eyes. Myopic eyes had thicker muscles than emmetropic eyes at nasal positions, except CM25 and CMT3, and at CM75 temporally. During accommodation and only nasally, CML reduced in emmetropic and myopic eyes, and CMLarc reduced in myopic eyes only. During accommodation, both nasally and temporally, muscles thickened anteriorly (CMT1 and CM25) and thinned posteriorly (CMT3 and CM75) except for temporal CM75. Moderate myopic eyes had greater temporal CMLarc than low myopic eyes, and the moderate myopes had thicker muscles both nasally and temporally using fixed and proportional distances.

**Conclusions:**

People with myopia had longer and thicker ciliary muscles than people with emmetropia. During accommodation, the anterior muscle thickened and the curved nasal muscle length shortened, more in myopic than in emmetropic eyes. The fixed distance method is recommended for repeat measures in the same individual. The proportional distance method is recommended for comparisons between refractive groups.

Knowledge of ciliary muscle morphology is important in both presbyopia^1^ and myopia research fields.^2^^–^^4^ However, imaging the ciliary body in vivo is hampered by its location behind the pigmented iris. Ultrasound biomicroscopy (UBM), a contact method, has been used to obtain in vivo images of the ciliary muscle.^5^^–^^7^ However, non-contact anterior segment optical coherence tomography (AS-OCT) produces high resolution magnified images and allows for easier identification of the scleral spur, which is a reference point for ciliary muscle measurements.^7^

The evidence for an association between ciliary muscle morphology and myopia is not consistent; some studies reported thicker ciliary muscle anteriorly in myopic eyes than in emmetropic eyes,^8^^–^^10^ whereas others found no relationship between anterior thickness and axial length.^10^^,^^11^ More recently, one study found that emmetropic eyes had thicker anterior (up to 1.4 mm from the scleral spur) and thinner posterior (1.4 to 4.5 mm from the scleral spur) ciliary muscles than myopic eyes.^12^

As a general finding, during accommodation the ciliary muscle thickens anteriorly and thins posteriorly,^8^^,^^11^^,^^13^ in both emmetropic and myopic eyes. Wagner et al.^12^ found that ciliary muscle thinned anteriorly (around 1 mm posterior to the scleral spur) and thickened posteriorly during accommodation, the latter being narrower in myopic eyes than in emmetropic eyes. Wagner et al.^14^ found that, following 30 minutes of reading at 25 cm, the ciliary muscle thinned anteriorly (0 to 1.4 mm) in emmetropic eyes and posteriorly (1.0 to 1.9 mm) in myopic eyes.

It has been suggested that ciliary muscle size (and presumably strength) may play a role in myopia development.^9^^,^^11^^,^^15^ Bailey et al.^9^ proposed that ciliary muscle thickening leads to poorer contractile responses and thus accommodative dysfunction (i.e. a reduced response, and hence a greater accommodation lag). This is the basis of the hyperopic defocus model, in which retinal defocus during near vision causes axial elongation and myopia.^16^^,^^17^ Another suggestion is that ciliary muscle tone affects the tension of the choroid^18^ and the increased muscle thickness alters the muscle mechanical properties,^19^ with both of these mechanical effects influencing the eye's axial length. Mutti suggested that a thicker ciliary muscle restricts equatorial expansion of the globe, leading to less crystalline lens thinning and lower power.^19^

The literature has information concerning a potential ciliary muscle mechanism for myopia development.^20^ One suggestion is that during accommodation, the ciliary muscle pulls on the smooth muscle of the choroid, mechanically thins the choroid, and produces the measured axial length increase. Another possibility is that a change to choroidal blood flow occurs with ciliary muscle contraction due to activation of the autonomic innervation. These theories that are proposed by Aggarwala^21^ and Logan et al.^20^ require investigation.

Previous AS-OCT studies have analyzed the ciliary muscle images using two methods: fixed distance measures^8^^,^^10^^,^^22^^,^^23^ and proportional distance.^11^^,^^24^ Thickness measurements taken at a fixed distance from the scleral spur do not take into account the fact that the muscle length varies significantly with eye size and refraction, so a point 2 mm from the scleral spur may represent an anatomically different region of the ciliary body in myopic eyes than in emmetropic eyes.^11^ Proportional thickness measures, such as 50% (CM50), of the curved muscle length would be more valid in analyzing participants of different refractive errors in terms of ensuring that similar regions of the ciliary muscle are compared. However, this method requires identification of the posterior end point of the ciliary muscle, which is poorly defined. To overcome the limitations of both analyses, Bailey^25^ suggested measuring the maximum thickness, which does not rely on the location of the scleral spur or the accurate identification of the posterior end point of the ciliary muscle.

Anatomic studies that may provide insight into differences in myopic and emmetropic eyes are important. There has been no study of the ciliary muscle dimensions using both fixed and proportional distance image analysis methods and maximum thickness in emmetropic and myopic eyes. This study will address this and investigate racial differences in ciliary muscle parameters. Anatomic differences that are associated with race might account for ethnic variations in myopia prevalence. Differences in the prevalence of myopia across races,^26^^,^^27^ particularly between East Asians and South Asians versus Caucasians, may be associated with the differences in ciliary muscle parameters.

Comparing the muscle dimensions with different methods during accommodation might help identify an appropriate method for future studies as well as exploring the ciliary muscle morphology during accommodation, which together could provide insights into physiological reasons for myopia development.

## Materials and Methods

### Participants

Seventy young adults (25 with emmetropia, and 45 with myopia) aged 18 to 27 years were recruited from Queensland University of Technology students and their colleagues. The participants were of Caucasian, East Asian (including Chinese, Japanese, and South Korean), and South Asian (including Indian, Nepalese, and Sri Lankan) races and an analysis based on this race classification was made. People with emmetropia and people with myopia had subjective spherical equivalent refraction (SER) between −0.25 diopters (D) and +1.00 D and between −6.00 D and −0.50 D, respectively. People with myopia were subdivided into those with low myopia (−0.50 D to >3.00 D) and those with moderate myopia (−3.00 D to −6.00 D). All participants had good ocular and general health, anisometropia ≤1.50 D, and cylinders ≤1.50 D.

Participants had comprehensive ophthalmic examinations that included slit-lamp biomicroscopy, direct ophthalmoscopy, non-cycloplegic automated refraction (Grand Seiko autorefractor WAM 5500), intraocular pressure measurement (iCare TA01i rebound tonometer), and non-cycloplegic distance subjective refraction. Best-corrected visual acuities were 0.0 logMAR (Snellen 6/6) or better. Subjective amplitude of accommodation was measured with a Rodenstock handheld Badal optometer (with a distance correction in place) at approximately 500 cd/m^2^ luminance.^28^ Participants were instructed to bring the target from far toward the eye and stop when the bottom line of 6/12 sized characters first became clear (far point), and then bring the target toward the eye until the same line became first unreadable (near point). The dioptric difference between the far and near points was the amplitude of accommodation. Assessment of ciliary muscle morphology was performed for the right eyes.

### Carl Zeiss Meditec Visante OCT Image Capture

The Carl Zeiss Meditec Visante (Dublin, CA, USA) uses low-coherence interferometry with a 1310 nm superluminescent light-emitting diode. The Visante OCT was set at high-resolution corneal mode for all images to provide an axial resolution of approximately 8 µm. The scanning plane was set at 0 degrees throughout the test. The Visante was modified to measure the ciliary muscle during accommodation. Accommodation (6 D) was induced using a simple Badal system attached to the Visante OCT as described by Laughton et al.^29^ The Badal system was built by suspending a +10.00 D lens and a Maltese cross target on a rotatable metal rod attached to the forehead rest. The Badal system was rotatable on either side of the instrument so that both the nasal and temporal regions of the ciliary muscle could be scanned. Images of ciliary muscle were taken when the eye was fixating an eccentrically positioned target at 40 degrees. The eye rotation, as compared with head turn, is necessary to maximize the palpebral aperture and therefore the OCT acquisition window. For myopic eyes with SER ≤ −2.00 D, daily disposal soft contact lenses (60% 1 day, Cooper Vision omafilcon A, Hamble, UK) were worn to increase the effective range of the Badal lens.

The image capture process lasted approximately 5 to 10 s per scan. Alignment of the device was achieved using the white light spot on the bulbar conjunctiva that corresponded to the section of the ciliary muscle being imaged. The white light was aligned at the screen center to ensure that images of the same section of the ciliary muscle were taken for all participants. The three best quality images, based on image clarity and thus identification of scleral spur and posterior end of the muscle, were selected. Averages of measures from the three images were used for statistical analysis.

### Ciliary Muscle Image Analysis

Laughton et al.^29^ validated an automatic measurement program for the ciliary muscle image analysis. The mean linear length and maximum thickness obtained from the software and the manual internal Visante calipers had good limits of agreement and inter-session repeatability. The software applies refractive indices to the scleral and ciliary muscle tissue of 1.41 and 1.38, respectively, in the y-direction. The posterior end of the ciliary muscle is identified as the point where the curves fitted to the inner and outer ciliary muscle borders reach a minimum separation.

**Figure 1.**
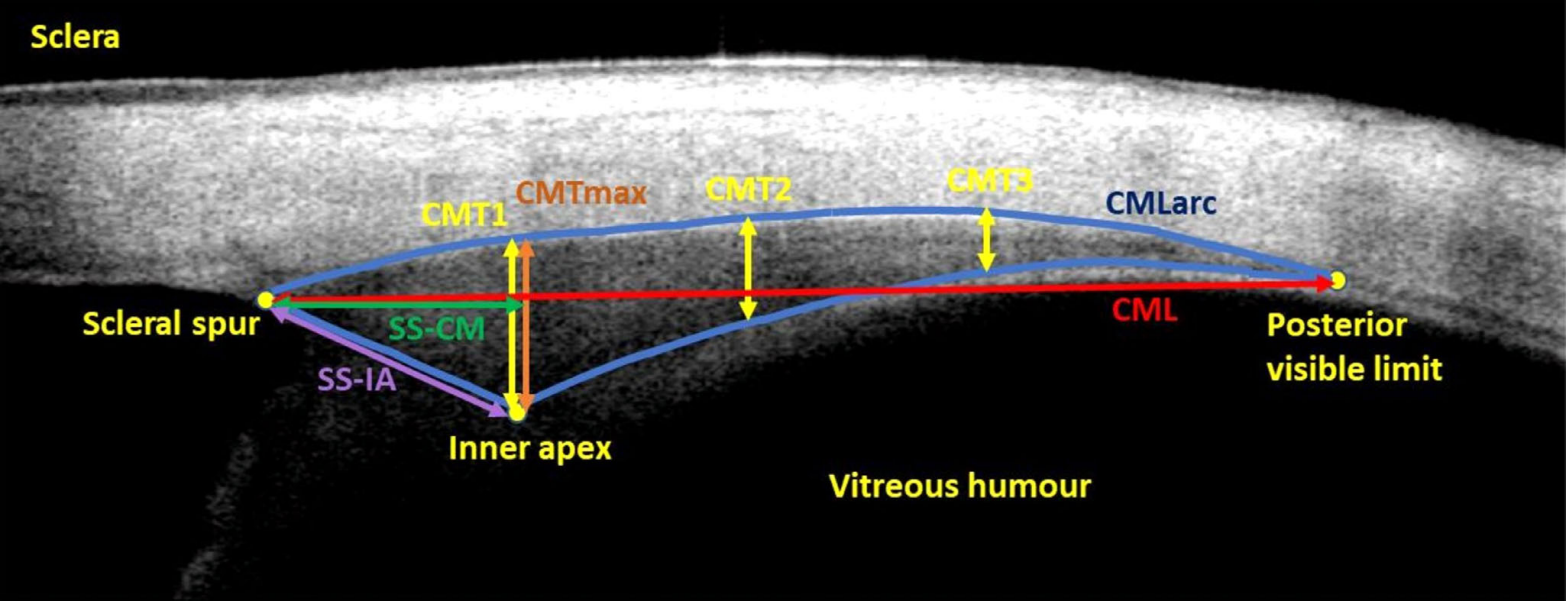
Visante OCT image of the unscaled nasal ciliary muscle. Distances shown are the linear (CML) and curved (CMLarc) lengths between the scleral spur and the posterior visible limit (*red and top blue lines*, respectively), maximum thickness (CMTmax, *orange line*), the anterior length from the scleral spur to CMTmax (SS-CM, *green line*), length from the scleral spur and inner apex (SS-IA, *purple line*), and thickness measurements at 1, 2, and 3 mm posterior to the scleral spur along the scleral curve (CMT1, CMT2, and CMT3, *yellow lines*). Proportional thicknesses CM25, CM50, and CM75 are not shown.

The software exports the following dimensions (see Fig. 1) to an Excel spreadsheet:

i. Ciliary muscle length (CML): the linear distance between the scleral spur and posterior visible limit,
ii. Ciliary muscle curve length (CMLarc): a curved-line ciliary muscle length along the scleral/ciliary muscle boundary,
iii. Maximum ciliary muscle thickness (CMTmax): maximum thickness regardless of position,
iv. Ciliary muscle length to the scleral spur (SS-CM): anterior length measured perpendicularly from the line of maximum thickness to the scleral spur,
v. SS-IA: the straight-line distance between the scleral spur and the inner apex,
vi. CMT1, CMT2, and CMT3: thicknesses at 1, 2, and 3 mm, respectively, from the scleral spur along the scleral curve, and
vii. CM25, CM50, and CM75: thicknesses at 25%, 50%, and 75%, respectively, of the curved length of the ciliary muscle.

### Data Analysis

Data analysis was performed using the Statistical Package for the Social Sciences (SPSS; version 26, IBM Statistics, New York, NY, USA). The level of significance was 0.05 for 2-tailed tests. The differences in characteristics between emmetropic and myopic eyes and between low myopic and moderate myopic eyes were tested using unpaired *t*-tests for numerical variables (age, SER, and axial length), as data were normally distributed according to Shapiro-Wilk tests, and chi-squared tests for categorical variables (gender, race, and parental history of myopia).

The differences in muscle dimensions either during accommodation or between refractive groups were presented as means and standard errors, whereas the rest of the parameters were presented as mean and standard deviations.

For both nasal and temporal regions, linear mixed models (LMMs) were used to investigate the differences in muscle dimensions between unaccommodated (0 D) and accommodated (6 D) states and to determine whether differences were dependent upon refraction group (emmetropia and myopia, and low and moderate myopia). The particular worth of the LMM analysis is that, if there is a missing data value for a particular position, this does not exclude the complete participant data set but uses all the data except the missing point in the analysis.

The LMMs included a random intercept with a variance components covariance and maximum likelihood estimation method. The associations between the muscle length and thicknesses and the demographic variables (age, gender, race, and family history), accommodation demands, and refractive status were first investigated by univariate linear mixed models. After this, variables meeting one or both of the following criteria were kept in a multivariate LMM: (i) variables with a significance level *P* < 0.20 in the univariate LMMs, (ii) variables which were different between emmetropia and myopia or between low and moderate myopia at *P* < 0.20 in the descriptive analysis (Table 1). Multiple models were assessed using a range of outcome variables, such as CML, CMT1, and CM25. This was adopted as there is no well-accepted single ciliary muscle parameter to study ciliary muscle dimension changes during accommodation across refractive groups. Previous studies have used a similar approach.^9^^,^^11^^,^^23^

**Table 1.** Characteristics of Emmetropic and Myopic Eyes

Characteristic	Overall (*n* = 70)	Emmetropia (*n* = 25)	Myopia (*n* = 45)	*P* Value	Low Myopia (*n* = 23)	Moderate Myopia (*n* = 22)	*P* Value
**Age, y**							
Mean ± SD	21.3 ± 2.5	21.2 ± 3.1	21.3 ± 2.5	0.88	20.8 ±2.7	21.7 ± 2.4	0.21
Range	18 to 27	18 to 27	18 to 27		18 to 27	18 to 27	
**Gender, female (%)**	41 (58.5)	17 (68.0)	24 (53.3)	0.83	10 (43.5)	14 (63.6)	0.13
**Race,** ^*^ ***n* (%)**							
Caucasian	12 (17.1)	7 (28.0)	5 (11.1)	**0.009** **^§^**	4 (17.4)	1 (4.5)	**0.002** **^§^**
East Asian	23 (32.9)	2 (8.0)	21 (46.7)		6 (26.1)	15 (68.2)	
South Asian	24 (34.2)	11 (44.0)	13 (28.9)		9 (39.1)	4 (18.2)	
Other	11 (15.8)	5 (20.0)	6 (13.3)		4 (17.4)	2 (9.1)	
**Family history of myopia, *n* (%)**							
Neither parent	21 (30.0)	15 (60.0)	6 (13.3)	**<0.001** **^§^**	6 (26.1)	0 (0)	**<0.001** ^§^
One parent^**^	31 (44.3)	7 (28.0)	24 (53.3)		11 (47.8)	13 (59.1)	
Both parents	18 (25.7)	3 (12.0)	15 (33.3)		6 (26.1)	9 (40.9)	
**SER, D**							
Mean ± SD	NA	+0.21 ± 0.36	−2.84 ± 1.72	**<0.001**	−1.43 ± 0.59	−4.31 ± 1.15	**0.004**
Range		−0.40 to +0.85	−0.50 to −5.83		−0.50 to −2.45	−3.84 to −5.83	
**Axial length, mm**							
Mean ± SD	NA	23.08±0.66	24.87±0.96	**<0.001**	24.33±0.96	25.41 ± 0.69	0.28
Range		21.96 to 24.64	21.97 to 26.96		21.96 to 25.86	23.59 to 26.96	
**Amplitude of accommodation, D**							
Mean ± SD	8.5 ± 1.1	8.3 ± 1.2	8.6 ± 1.1	0.24	8.5 ± 1.3	8.7 ± 0.9	0.15
Range	6.25 to 10.0	6.25 to 10.0	6.0 to 10.0		6.0 to 9.75	7.0 to 10.0	

SD, standard deviation.

* East Asian consisted of 20 Chinese, 2 Japanese and 1 South Korean; South Asian consisted of 14 Nepalese, 9 Indians and 1 Sri Lankan; Other category consisted of 3 Vietnamese, 3 Indonesians, 3 Mixed, 1 Filipino, and 1 Malay.

** Includes 3 participants with a myopic sibling.

§ Chi-squared test; NA, not applicable. Significant comparisons (i.e. *P* < 0.05) are bolded.

For both nasal and temporal regions, the backward fitting approach was performed, that is, the variable with the highest *P* value was excluded from the model until the model was left with variables that were significant. For pairwise comparisons between races, the Sidak test was performed. The distributions of outcome variables were normal as assessed by graphical (histogram and normality plot) and quantitative (skewness) tests. Distributions of the residuals were assessed using histogram normal probability plots and were normally distributed. Image data of three people (one emmetropic and two low myopic) eyes were excluded for temporal analyses because of poor image quality.

## Results

There were 70 participants, with a mean age of 21.3 ± 2.5 years and 41 (58.5%) women (see Table 1). For emmetropia (*n* = 25) and myopia (*n* = 45), the mean SER of the right eyes were +0.21 ± 0.36 D and −2.84 ± 1.72 D, respectively. For low myopia (*n* = 23) and moderate myopia (*n* = 22), axial lengths were 24.3 ± 1.0 mm and 25.4 ± 0.7 mm, respectively. The mean amplitude of accommodation across all participants was 8.5 ± 1.1 D. There were significant differences in the distribution of race between emmetropes and myopes (χ^2^ test, *P* = 0.009): emmetropes were primarily South Asians (11, 44%) and Caucasians (7, 28%), whereas myopes were primarily East Asians (21, 47%) and South Asians (13, 29%). Fifteen (60%) of people with emmetropia reported having no parent with myopia and 28% (7) one parent with myopia, whereas 53% (24) of people with myopia reported one parent with myopia and 33% (15) had two parents with myopia (χ^2^ test, *P* < 0.001).

The rest of this Results section describes the ciliary muscle dimensions and how these were affected by refraction group and accommodation. Table 2 summarizes the ciliary muscle dimension changes that occurred with accommodation.

**Table 2.** Comparison of Ciliary Muscle Dimensions in Emmetropia and Myopia for Both Nasal and Temporal Regions

Dimensions	Nasal	Temporal
CML	MYP > EMM, mean difference = 303 ± 76 µm, F_1,70_ = 1.84, *P* = 0.18. Correlated positively with AL (unaccommodated state, *r* = 0.40, *P* < 0.001 and accommodated state, *r* = 0.33, *P* = 0.005). Decreased with accommodation, mean change = 82 ± 28 µm, F_1,70_ = 8.34, *P* = 0.005.	No significant difference between EMM and MYO, mean difference = 114 ± 84 µm, F_1,67_ = 15.80, *P* = 0.001. Correlated positively with AL (unaccommodated state, *r* = 0.24, *P* = 0.049 and accommodated state, *r*= 0.26, *P* = 0.039). No change with accommodation, mean change = 26 ± 34 µm, F_1,67_ = 0.58, *P* = 0.45.
CMLarc	MYO > EMM, mean difference = 334 ± 80 µm, F_1,70_ = 17.56, *P* < 0.001. Interaction between refractive status and accommodation: MYO had shorter length during accommodation, but EMM had no significant difference (126 ± 36 vs. 4 ± 48 µm, *P* = 0.03). Correlated positively with AL (unaccommodated state, *r* = 0.41, *P* < 0.001 and accommodated state, *r* = 0.33, *P* = 0.005).	No significant difference between EMM and MYO, mean difference = 112 ± 87 µm, F_1,67_ = 1.67, *P* = 0.20. No change with accommodation, mean change = 34 ± 35 µm, F_1,67_ = 0.92, *P* = 0.34. No correlation with AL (for unaccommodated and accommodated states, *r* = 0.24, *P* = 0.05).
SS-CM	17.5% of the total muscle in EMM and MYO. No correlation with AL (*r* =0.08, *P* = 0.30).	15.0% of the total muscle in EMM and MYO. Correlated positively with AL (*r*= 0.28, *P* = 0.001).
CMT1	MYO > EMM, mean difference = 81 ± 36 µm, F_1,70_ = 5.13, *P* = 0.03. Thicker with accommodation by 50 ± 6 µm, F_1,70_ = 58.17, *P* < 0.001. Correlated positively with AL (*r* = 0.25, *P* = 0.03).	No significant difference between EMM and MYO, mean difference = 47 ± 40 µm, F_1,67_ = 1.37, *P* = 0.25. Thicker with accommodation by 50 ± 6 µm, F_1,67_ = 58.17, *P* < 0.001. No correlation with AL (*r* = 0.15, *P* = 0.21).
CMT2	MYO > EMM, mean difference = 79 ± 28 µm, F_1,70_ = 7.94, *P* = 0.006. No change with accommodation, mean change = 7 ± 6 µm, F_1,70_ = 1.09, *P* = 0.29.	No significant difference between EMM and MYO, mean difference = 54 ± 28 µm, F_1,67_ = 3.87, *P* = 0.05. No change with accommodation, mean change = 6 ± 5 µm F_1,67_ = 1.60, *P* = 0.21.
CMT3	MYO > EMM, mean difference = 73 ± 21 µm, F_1,70_ = 12.34, *P* = 0.001. Thinner with accommodation by 24 ± 4 µm, F_1,70_ = 35.58, *P* < 0.001. Correlated positively with AL (*r* = 0.37, *P* = 0.002).	MYO > EMM mean difference = 45 ± 21 µm, F_1,67_ = 4.60, *P* = 0.04. Thinner with accommodation by 10 ± 4 µm, F_1,67_ = 4.68, *P* = 0.03. Correlated positively with AL (*r* = 0.27, *P*= 0.03).
CMTmax	No significant difference between EMM and MYO, mean difference = 44 ± 44 µm, F_1,70_ = 0.97, *P* = 0.32. Increased with accommodation by 69 ± 11 µm, F_1,70_ = 42.06, *P* < 0.001. No correlation with AL (*r* = −0.02, *P* = 0.87).	No significant difference between EMM and MYO, mean difference = 28 ± 52 µm, F_1,67_ = 0.28, *P* = 0.59. Increased with accommodation by 74 ± 15 µm, F_1,67_ = 23.83, *P* < 0.001. No correlation with AL (*r* = −0.03, *P* = 0.82).
SS-IA	MYO > EMM, mean difference = 117 ± 54 µm, F_1,70_ = 4.58, *P* = 0.04. Increased with accommodation by 61 ± 13 µm, F_1,70_ = 20.45, *P* < 0.001.	No significant difference between EMM and MYO, mean difference = 117 ± 54 µm, F_1,67_ = 0.58, *P* = 0.45. No change with accommodation, mean change = 22 ± 20 µm, F_1,67_ = 1.22, *P* = 0.27.
CM25	No significant difference between EMM and MYP, mean difference = 63 ± 35 µm, F_1,70_ = 3.24, *P* = 0.07. Increased with accommodation by 53 ± 8 µm, F_1,70_ = 47.94, *P* < 0.001. Correlated positively with AL (*r* = 0.22, *P* = 0.009).	No significant difference between EMM and MYO, 44 ± 38 µm, F_1,67_ = 1.34, *P* = 0.35). Increased with accommodation by 48 ± 7 µm, F_1,67_ = 46.00, *P* < 0.001. No correlation with AL (*r* = 0.10, *P* = 0.24).
CM50	MYO > EMM, mean difference = 60 ± 24 µm, F_1,70_ = 6.06, *P* = 0.01. No significant difference with accommodation, mean difference = 8 ± 7 µm, F_1,70_ = 1.09, *P* = 0.29.	No significant difference between EMM and MYO, mean difference = 37 ± 22 µm, F_1,67_ = 2.88, *P* = 0.09. No significant difference with accommodation, mean difference = 6 ± 6 µm, F_1,67_ = 0.96, *P* = 0.33.
CM75	MYO > EMM, mean difference = 35 ± 13 µm, F_1,70_ = 7.05, *P* = 0.01. Decrease with accommodation by 53 ± 8 µm, F_1,70_ = 47.94, *P* < 0.001.	No significant difference between EMM and MYO, mean difference =15 ± 19 µm, F_1,67_ = 0.56, *P* = 0.46. No change with accommodation, mean difference = 10 ± 15 µm, F_1,67_ = 0.44, *P* = 0.50.
CMTmax and race	No significant effect	Thicker with East Asians (mean difference = 148 ± 68 µm, *P* = 0.03) and South Asians (mean difference = 203 ± 69 µm, *P* = 0.006) than with Caucasians.
CM25 and race	No significant effect	East Asians (mean difference = 122 ± 51 µm, *P* = 0.03) and South Asians (mean difference = 125 ± 52 µm, *P* = 0.03) had thicker temporal CM25 than Caucasians.

MYO, myopia; EMM, emmetropia; AL, axial length.

### Lengths

**Figure 2.**
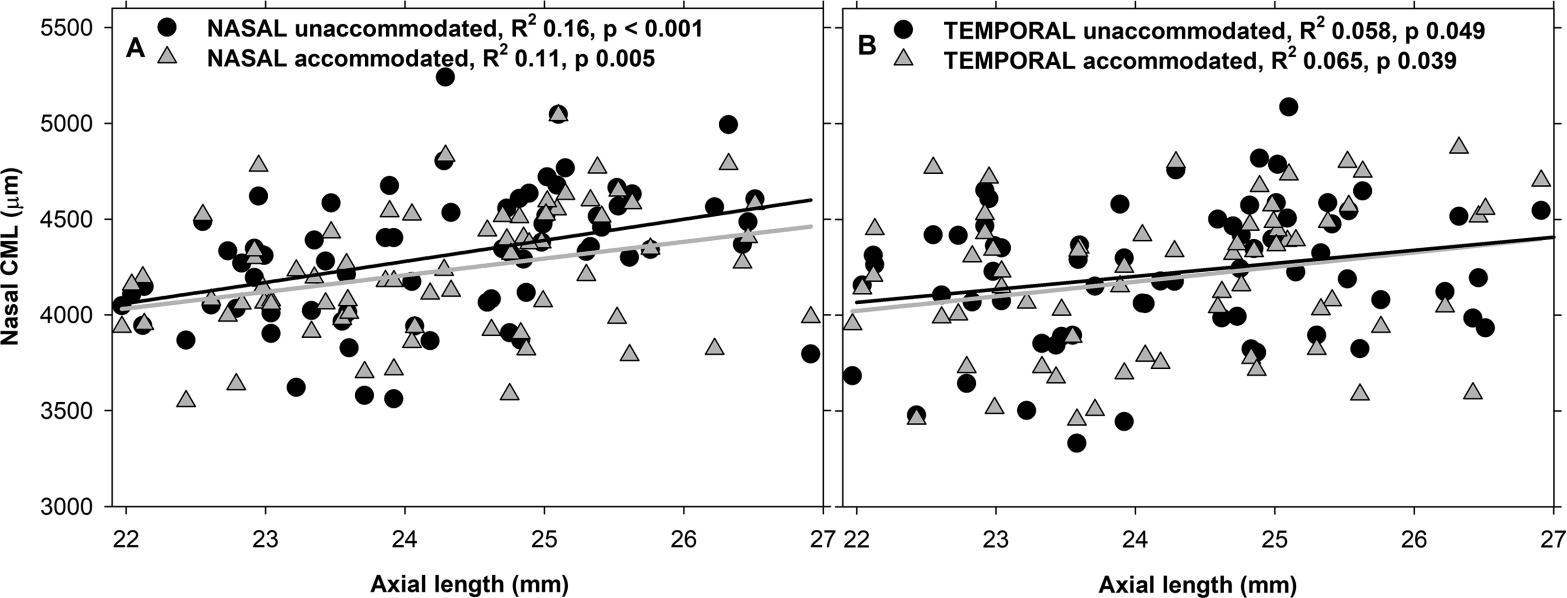
Scatter plot of data of all participants for linear ciliary muscle length (CML) and axial length in unaccommodated and accommodated states in the nasal (**A**) and temporal (**B**) regions.

In the unaccommodated state, myopic eyes had greater CML than emmetropic eyes in the nasal region (mean difference = 303 ± 76 µm, F_1,70_ = 15.80, *P* = 0.001). CML shortened nasally during accommodation (mean change = 82 ± 28 µm, F_1,70_ 8.34, *P* = 0.005), with no statistical difference between myopes and emmetropes (120 ± 35 vs. 14 ± 47 µm, *P* = 0.07). Both nasal and temporal CML were correlated positively with the axial length in the unaccommodated state and accommodated states (Fig. 2).

In the unaccommodated state, myopic eyes had greater CMLarc than emmetropic eyes in the nasal region (mean difference = 334 ± 80 µm, F_1,70_ = 17.56, *P* < 0.001). CMLarc shortened during accommodation in the nasal region (mean change = 80 ± 29 µm, F_1,70_ = 7.30, *P* = 0.009), with myopic eyes having greater reduction than emmetropic eyes (126 ± 36 vs. 4 ± 48 µm, *P* = 0.03). Nasal CMLarc was correlated positively with the axial length for both unaccommodated (*r* = 0.41, *P* < 0.001) and accommodated (*r* = 0.33, *P* = 0.005) states.

In the unaccommodated state, myopic and emmetropic eyes had similar SS-CM both nasally (mean difference = 38 ± 144 µm, F_1,70_ = 0.07, *P* = 0.80) and temporally (15 ± 91 µm, F_1,70_ = 0.03, *P* = 0.87). In the unaccommodated state, SS-CM was correlated positively with the axial length in the temporal region (*r* = 0.28, *P* = 0.001).

In the unaccommodated state, myopic eyes had longer SS-IA than emmetropic eyes in the nasal region (mean difference = 117 ± 54 µm, F_1,70_ = 4.58, *P* = 0.04). SS-IA increased during accommodation only nasally (61 ± 13 µm, F_1,70_ = 20.45, *P* < 0.001).

### CMTmax

In the unaccommodated state, myopic and emmetropic eyes had similar CMTmax in both nasal (mean difference = 44 ± 44 µm, F_1,70_ = 0.97, *P* = 0.32) and temporal (28 ± 52 µm, F_1,67_ = 0.28, *P* = 0.59) regions. The CMTmax thickened during accommodation both nasally (mean change = 69 ± 11 µm, F_1,70_ = 42.06, *P* < 0.001) and temporally (74 ± 15 µm, F_1,67_ = 23.83, *P* < 0.001).

### Fixed Distance Analysis Method for Thicknesses

In the unaccommodated state, myopic eyes had greater CMT1 than emmetropic eyes in the nasal region (mean difference = 81 ± 36 µm, F_1,70_ = 5.13, *P* = 0.03). CMT1 thickened during accommodation in both nasal (mean change = 50 ± 6 µm, F_1,70_ = 58.17, *P* < 0.001) and temporal (48 ± 5 µm, F_1,67_ = 81.85, *P* < 0.001) regions. Nasal CMT1 was correlated positively with axial length for both unaccommodated and accommodated states, but the temporal CMT1 was not for either accommodation state.

In the unaccommodated state, myopic eyes had greater CMT2 than emmetropic eyes in the nasal region (mean difference = 79 ± 28 µm, F_1,70_ = 7.94, *P* = 0.006). CMT2 did not change significantly during accommodation either nasally (mean = 7 ± 6 µm, F_1,70_ = 1.09, *P* = 0.29) or temporally (6 ± 5 µm F_1,67_ = 1.60, *P* = 0.21).

In the unaccommodated state, myopic eyes had greater CMT3 than emmetropic eyes for both nasal (mean difference = 73 ± 21 µm, F_1,70_ = 12.34, *P* = 0.001) and temporal (45 ± 21 µm, F_1,67_ = 4.60, *P* = 0.04) regions. CMT3 thickened during accommodation both nasally (mean change = 24 ± 4 µm, F_1,70_ = 35.58, *P* < 0.001) and temporally (10 ± 4 µm, F_1,67_ = 4.68, *P* = 0.03).

### Proportional Analysis Method for Thicknesses

In the unaccommodated state, myopic and emmetropic eyes had similar CM25 in both nasal (mean difference = 63 ± 35 µm, F_1,70_ = 3.24, *P* = 0.07) and temporal (44 ± 38 µm, F_1,67_ = 1.34, *P* = 0.35) regions. CMT25 thickened during accommodation both nasally (mean change 53 ± 8 µm, F_1,68_ = 47.94, *P* < 0.001) and temporally (48 ± 7 µm, F_1,67_ = 46.00, *P* < 0.001). CM25 was correlated positively with the axial length in the nasal region (*r* = 0.22, *P* = 0.009), but not in the temporal region (*r* = 0.10, *P* = 0.24).

In the unaccommodated state, myopic eyes had greater CM50 than emmetropic eyes in the nasal region (mean difference 60 ± 24 µm, F_1,70_ = 6.06, *P* = 0.01). CM50 did not change significantly during accommodation either nasally (8 ± 7 µm, F_1,70_ = 1.09, *P* = 0.29) or temporally (6 ± 6 µm, F_1,67_ = 0.96, *P* = 0.33).

In the unaccommodated state, myopic eyes had greater CM75 than emmetropic eyes in the nasal region (mean difference 35 ± 13 µm, F_1,70_ = 7.05, *P* = 0.01). CM75 thinned during accommodation nasally (53 ± 8 µm, F_1,68_ = 47.94, *P* < 0.001) and was not statistically significantly different between emmetropes and myopes (20 ± 8 vs. 8 ± 6 µm F_1,70_ = 1.57, *P* = 0.26).

**Figure 3.**
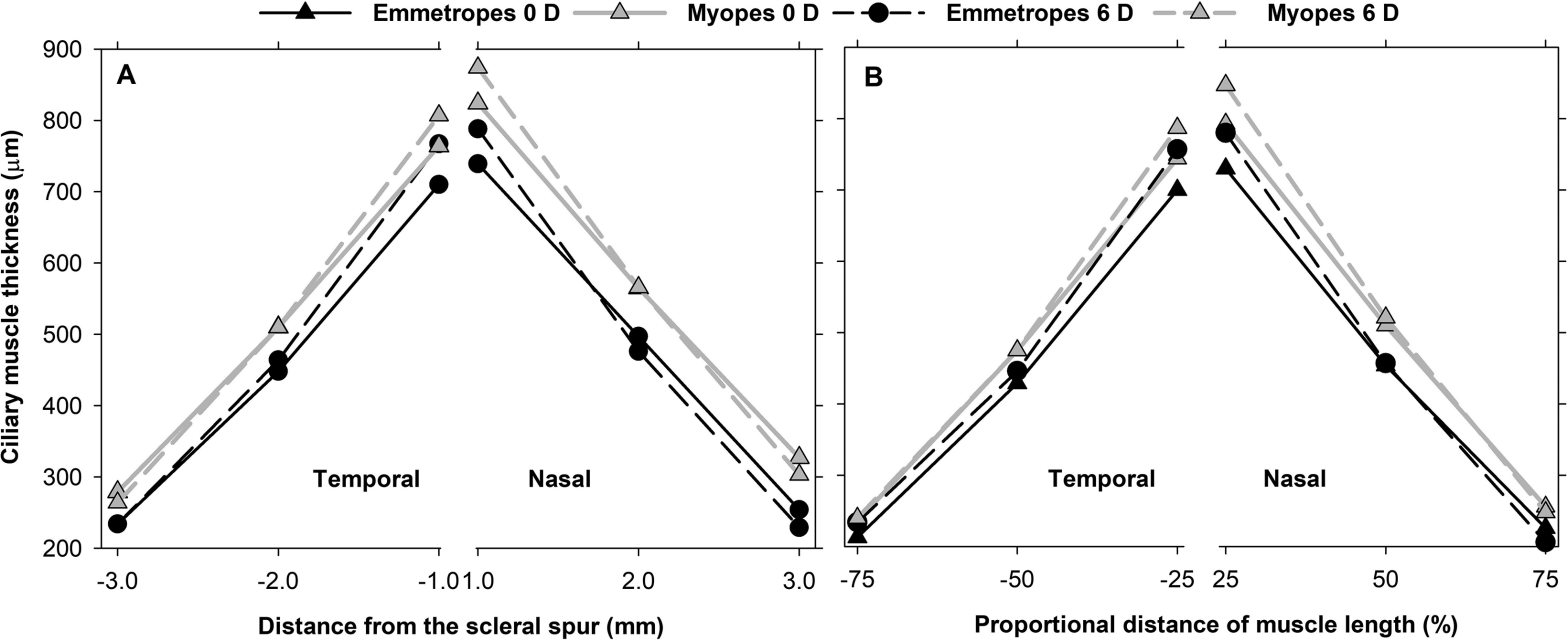
Ciliary muscle thickness in emmetropes and myopes, at unaccommodated (0 D) and accommodated (6 D) states in nasal and temporal regions using fixed distance (**A**) and proportional distance (**B**) methods. Black and gray colors represent emmetropic eyes and myopic eyes, respectively, and linear and dotted lines represent unaccommodated and accommodated states, respectively. Myopic eyes had thicker muscles than emmetropic eyes for both methods at all positions. During accommodation, ciliary muscle thickened anteriorly and thinned posteriorly on both regions for both methods except for temporal CM75.

Figure 3 is a summary of ciliary muscle thickness for emmetropic and myopic eyes using both fixed and proportional analysis methods.

### Dimensions and Race

**Figure 4.**
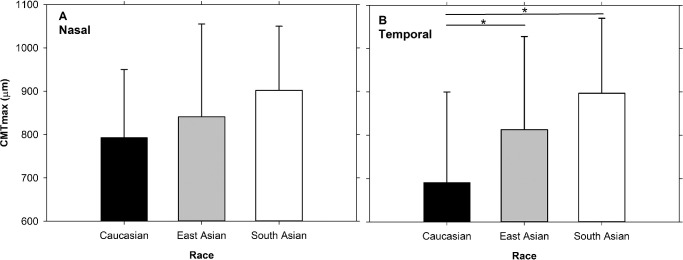
The maximum muscle thickness (CMTmax) for different races at nasal (**A**) and temporal (**B**) regions. Data presented as mean ± SD. *Indicates significant difference between groups.

In the unaccommodated state, nasal CMTmax was not different (F_2,70_ = 1.72, *P* = 0.19) across races, but temporal CMTmax was greater in East Asian (mean difference = 122 ± 68 µm, *P* = 0.03) and South Asian (206 ± 69 µm, *P* = 0.006) eyes than Caucasian eyes (Fig. 4).

### Dimensions in Low and Moderate Myopia

**Figure 5.**
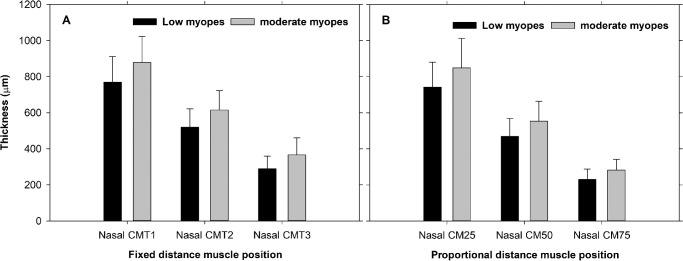
Nasal ciliary muscle thicknesses in low and moderate myopes using fixed (**A**) and proportional (**B**) distance method analyses. Data presented as means ± SD.

**Figure 6.**
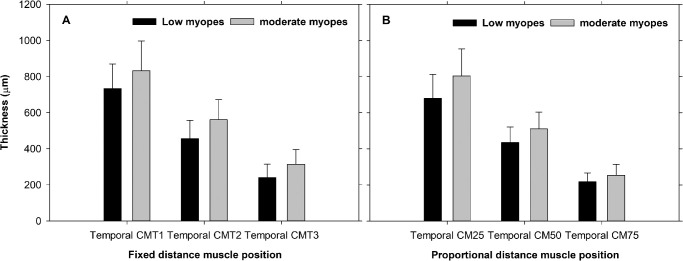
Temporal ciliary muscle thicknesses in low and moderate myopes using fixed (**A**) and proportional (**B**) distance method analyses. Data presented as means ± SD.

In the unaccommodated state, moderate myopic eyes had greater CMLarc than low myopic eyes temporally (mean difference = 222 ± 100 µm, F_1,43_ = 4.73, *P* = 0.03). Moderate myopic eyes had thicker muscle than low myopic eyes in all positions using both fixed and proportional analysis methods (Figs. 5, 6). In both myopic groups, the ciliary muscle thickened anteriorly (CMT1 and CM25) and thinned posteriorly (CMT3 and CM75) during accommodation both nasally and temporally using both the fixed and proportional analysis methods.

## Discussion

The dimensions of nasal and temporal ciliary muscle were measured from Visante OCT images at two accommodation states in young adult people with emmetropia and both low and moderate myopia, using fixed and proportional distance analyses. For the nasal region, myopic eyes had longer and thicker ciliary muscles than emmetropic eyes. With accommodation, the muscle shortened nasally more in myopic eyes than in emmetropic eyes, and it thickened anteriorly, both nasally and temporally, for both refractive groups. Both the muscle lengths and the nasal anterior muscle thickness were correlated positively with axial length, indicating longer and thicker muscles in larger eyes. Furthermore, the temporal maximum muscle thickness and the anterior muscle thickness determined using the proportionate method were greater in East Asian and South Asian eyes than those of Caucasian eyes; this was not an axial length effect as it was not influenced by race. Moderate myopic eyes had greater temporal CMLarc than low myopic eyes. For both myopic groups, the ciliary muscle thickened anteriorly and thinned posteriorly during accommodation both nasally and temporally.

These findings are consistent with previous studies that report greater ciliary muscle length and thicker anterior ciliary muscle in myopia than in emmetropia.^8^^–^^10^ The curved ciliary muscle lengths were shorter in our study than that of Sheppard and Davies.^11^ They reported mean unaccommodated muscle length of their participants (combined emmetropia and myopia) for nasal and temporal regions of 4.63 mm and 4.81 mm, respectively (i.e. with the larger length temporally). The corresponding results here were 4.26 mm and 4.23 mm. We found that the ciliary muscle was slightly longer temporally (temporal = 4.19 versus nasal = 4.15 mm) for emmetropic eyes, but slightly longer nasally for myopic eyes (temporal = 4.26 versus nasal = 4.36 mm).

Furthermore, the thicknesses were larger than reported by Sheppard and Davies^11^ and Buckhurst et al.^10^ for adults, and smaller than reported by Bailey et al.^9^ for children. For example, unaccommodated nasal CMT2 of our participants (combined emmetropia and myopia) was 531 µm, whereas the corresponding values were 347 µm for Sheppard and Davies,^11^ 313 µm for Buckhurst et al.,^10^ and 602 µm for Bailey et al.^9^ If Bailey et al.'s results are corrected for refractive index (they used 1.0 rather than 1.382), their mean result is 435 µm, which is now smaller than ours.

A potential reason for these differences in thickness across studies is related to the study participants. Buckhurst et al.^10^ had predominately Caucasians (62%) followed by South Asian (38%) participants, whereas we had predominately South Asians (34%) and East Asians (33%) followed by Caucasian (17%) participants. South Asians and East Asians had greater CMTmax and CM25 for the temporal region than their Caucasian counterparts here, whereas South Asians had greater CMT1, CMT2, and CMT3 than Caucasians for the nasal region in Buckhurst et al.^10^ The differences in muscle morphology may be linked to a larger prevalence of myopia in the East Asian and South Asian races than in the Caucasian race.^30^^,^^31^

In line with previous studies,^8^^,^^11^^,^^13^ accommodation caused length shortening, anterior ciliary muscle thickening, and posterior muscle thinning. These findings support the generally accepted model of ciliary muscle action during accommodation, whereby most of the muscle mass shifts anteriorly and inward to reduce zonular tension.^32^^,^^33^ There might be a link between the thicker anterior ciliary muscle and the reduced accommodation response during near activities in myopia. It is believed that alterations in ciliary muscle dimensions are due to muscle hypertrophy which would result in inadequate ciliary muscle contraction^9^^,^^34^ and may explain the accommodative lags found in myopic children.^35^^,^^36^ Our findings that moderate myopic eyes had thicker ciliary muscle than low myopic eyes supported the link between the refractive status and muscle dimensions, where the greater the myopia, the thicker the ciliary muscle.

A recent study has found a contrasting result to our study and earlier studies, with emmetropic eyes having thicker anterior ciliary muscle up to 1.4 mm from the scleral spur than myopic eyes.^12^ The reason for this contrasting result is not known.

Here, ciliary muscle dimensions were measured using both fixed^8^^,^^10^^,^^22^ and proportional distance^11^^,^^24^ analysis methods. The fixed distance analysis does not take into account the variation of the muscle length due to refractive error, and a point 2 mm from the scleral spur may represent an anatomically different region of the ciliary muscle in myopic eyes than in emmetropic eyes.^11^ This issue is addressed by the proportional analysis, where the thickness is measured based on the proportion of the muscle length, but this requires accurate identification of the ciliary muscle's posterior limit.

It is of interest as to whether ciliary muscle asymmetries may affect myopia development and progression or influence retinal contour asymmetry. Asymmetry findings are mixed. Sheppard and Davies^11^ found no difference in the length between temporal and nasal regions, but found greater temporal than nasal thickness at CM50 and CM75 positions. Zhang et al.^37^ found the ciliary muscle was longer and thicker temporally than nasally. We found the muscle was longer temporally than nasally in emmetropic eyes, but thickness was greater nasally than temporally for both emmetropic and myopic eyes. Sheppard and Davies^11^ suggested nasal/temporal ciliary muscle differences could give a stronger contractile response on the side where the muscle is thickest. Beyond any potential implications for myopia, nasal/ temporal variation in ciliary muscle morphology and contractile response may have implications in surgical and pharmaceutical strategies to restore accommodation to presbyopic eyes. Nasal/ temporal anatomic variations may be a functional necessity in primates to enable best alignment of the lenticular axes to maintain binocular single vision during eye movements that accompany accommodation.^38^

We propose that the fixed distance analysis is preferable for comparing changes in muscle thickness due to accommodation or to myopia onset. As this method does not require identification of the muscle's posterior end point, any errors related to that measurement would be eliminated, resulting in more accurate repeated measures. Conversely, proportional distance analysis is preferable for comparing the muscle dimensions between the refractive groups, such as emmetropia versus myopia, as this method compares similar regions of the muscle in groups where eye sizes might differ. Recently, Wagner et al.^39^ and Straßer et al.^40^ developed a semi-automatic segmentation program that allows fine measurement of thicknesses of ciliary muscle.

In terms of study limitations, it is likely that some participants did not accommodate fully during the task, and we were not able to make simultaneous measurement of refraction and ciliary muscle imaging to know the per diopter effect of accommodation. Whereas the sequential approach used here does not enable us to timelock biometric and refractive measures, it does provide an accommodative profile for each participant. Furthermore, to visualize the complete ciliary muscle, participants were required to view the target positioned eccentrically at 40 degrees to the instrument axis. For some participants, accurate fixation at this position was difficult to maintain. Finally, as the study included only adults, it limits interpretation of understanding how ciliary muscle may play a role in myopia development in children.

In conclusion, people with myopia had longer and thicker ciliary muscles than people with emmetropia. During accommodation, the anterior ciliary muscle thickened, and the curved nasal length shortened more in myopia than in emmetropia. The fixed distance analysis method is recommended when determining within-subject variation and the proportional distance analysis method is recommended when comparing refractive groups.
